# Minority M184V variants in women exposed to 3TC/FTC-containing lopinavir-ritonavir (LPVr) regimens in pregnancy

**DOI:** 10.1186/1758-2652-13-S4-P159

**Published:** 2010-11-08

**Authors:** S Surah, S Oshea, S Costelloe, M Hanlon, J Mullen, I Chrystie, GP Taylor, A De Ruiter

**Affiliations:** 1 Guy's & St Thomas' NHS Foundation Trust, Department of GUM & HIV, London, UK; 2 GSTS Pathology, Department Infection & Immunity, Virology Section, London, UK; 3 Guy's & St Thomas' NHS Foundation Trust, Directorate of Infection, London, UK; 4 Imperial College, Department of Infectious Diseases, London, UK

## Purpose of the study

Short-term antiretroviral therapy (START) consisting of 2 NRTIs and a ritonavir boosted PI is widely used in pregnancy. Resistance is rarely detected using population based sequencing. The aim of this study was to determine whether minority M184V variants emerge with 3TC/FTC containing START regimens in pregnancy and if so whether this impacted upon future treatment outcomes.

## Methods

Multi-centre study. An allele-specific real time PCR (ASPCR), optimized for subtypes B, C and AG, was used to detect minority M184V variants. Participants took START for the prevention of HIV mother-to-child transmission (PMTCT). All received standard dose LPVr. Plasma samples were tested pre and post treatment. Routine population based sequencing was also performed.

## Summary of results

ASPCR failed in 13/31 (42%) women. Among the remaining 18, 11(61%) were wild type (WT) and 7(22.5%) had minority M184V sequences (range of detection 0.5 to 14%). All samples were WT with population based sequencing. ASPCR failed to amplify from pre-treatment samples in 4/7 women with minority M184V and was WT in 3. Table 1 compares women with and without M184V mutants detected by ASPCR. Therapeutic drug monitoring (TDM) was performed in a subset.

**Table 1:** 

	M184V mutants N=7	WT N=11
Median Age, yrs (range)	30 (26.5-37)	34 (21-38)

African origin	7 (100%)	7 (64%)

European origin	0	2 (18%)

Caribbean origin	0	1 (9%)

South American origin	0	1 (9%)

Median gestation at START initiation, completed weeks (range)	22 (19-26)	21 (15-29)

Median duration START, days (range)	115 (87-132)	121 (68-151)

Virological suppression at delivery	6 (86%) (other VL 57c/ml)	11 (100%)

Subtype B	1 (14%)	4 (36%)

Subtype C	5 (72%)	1 (9%)

Subtype AG	1 (14%)	6 (55%)

Previous ART	2/7 (29%)	3/11 (27%)

Previous ART regimens	ZDVm, CBV/LPVr	ZDVm, CBV/nelfinavir, CBV/nelfinavir

Population based sequencing performed	7/7 (100%)	10/11 (91%) (1 failed)

WT on population based sequencing	7/7	10/10

Number of Lopinavir TDM performed	6/7 (86%)	5/11 (45%)

Median Lopinavir concentration (µg/L) (range)	4446 (2791-7551)	2991 (2023-7782)

Subsequent ART	4/7 (57%)	3/11 (27%)

Virological suppression with subsequent ART	4/4 (100%)	2/3 (67%) (1 stopped after a few weeks)

## Conclusions

M184V mutants were detected after LPVr based START for PMTCT in 22.5% women. No difference in prior ART, START duration, drug concentration or virological suppression was observed. The presence of minority M184V variants post-partum did not affect future treatment success. The possible association with HIV-1 subtype C requires further evaluation but clade effect on the development of resistance has been reported following intra-partum nevirapine.

